# A novel AAA+ ATPase required for sporulation and stress response in *Bacillus anthracis*

**DOI:** 10.1128/jb.00518-25

**Published:** 2026-02-19

**Authors:** Nitika Sangwan, Ankur Bothra, Andrei P. Pomerantsev, Aakriti Gangwal, Rasem Fattah, Mahtab Moayeri, Qian Ma, Sundar Ganesan, Chetkar Chandra Keshavam, Renu Baweja, Uma Dhawan, Stephen H. Leppla, Yogendra Singh

**Affiliations:** 1 Department of Biomedical Science, Bhaskaracharya College of Applied Sciences, University of Delhi 28742, New Delhi, India; 2 Microbial Pathogenesis Section, Laboratory of Parasitic Diseases, National Institute of Allergy and Infectious Diseases 35037, Bethesda, Maryland, USA; 3 Department of Zoology, University of Delhi 28742, New Delhi, India; 4 Biological Imaging Section, Research Technologies Branch, National Institutes of Allergy and Infectious Diseases, National Institutes of Health 2511, Bethesda, Maryland, USA; 5 Department of Biochemistry, Shivaji College, University of Delhi 123522, New Delhi, India; 6 Delhi School of Public Health, Institution of Eminence, University of Delhi 28742, New Delhi, India; The Ohio State University, Columbus, Ohio, USA

**Keywords:** AAA+ ATPase, BA PrkA, *Bacillus anthracis*, sporulation, osmotic stress, anthrax

## Abstract

AAA+ proteins function as molecular machines that utilize ATP to perform diverse cellular functions, including protein homeostasis, stress regulation, and cell cycle/developmental processes. In this study, we identified a novel AAA+ ATPase-related *Bacillus anthracis* protein BAS0518 (BA PrkA) which has 88% amino acid homology to *Bacillus subtilis* PrkA. Conserved domain analysis showed that BA PrkA has an N-terminal AAA+ ATPase domain with characteristic Walker A and Walker B motifs and a conserved secondary region of homology (SRH) domain, along with a C-terminal cAMP-dependent protein kinase domain. Based on AlphaFold 3 predicted structures, we classified BA PrkA as part of Clade III of the AAA+ superfamily. Our results suggest that BA PrkA has negligible protease and kinase activities under *in vitro* conditions. Nonetheless, BA PrkA was found to play a significant role in sporulation. It is temporally expressed during sporulation Stages II to VI. A null mutant lacking BA PrkA exhibits severe sporulation defects, with reduced spore viability and downregulation of genes related to spore integuments formation. These defects were reversed in a complementation strain expressing BA PrkA ectopically. Additionally, the null mutant strain showed compromised growth under ionic-osmotic stress conditions and showed a 13-fold induction of BA *prkA* in wild-type *B. anthracis* when exposed to 1 M NaCl. Analysis of the BA PrkA interactome revealed enrichment of two proteins, ProA and EzrA, which are implicated in osmotic stress response and the sporulation process, respectively. These findings show that BA PrkA is essential for sporulation and resistance to osmotic stress.

**IMPORTANCE:**

Sporulation and stress adaptation are vital for the survival of *Bacillus anthracis* in harsh environments. We characterize a novel AAA+ ATPase, *B. anthracis* BAS0518 (BA PrkA), which shares 88% similarity with *Bacillus subtilis* PrkA. Analysis of BA PrkA null mutant shows that BA PrkA is essential for proper spore development and stress resilience. BA PrkA is expressed during sporulation Stages II–VI, and its deletion causes defective, less viable spores, reduced osmotic stress tolerance, and downregulation of key sporulation genes. Interaction analyses identified ProA and EzrA, proteins linked to stress response and sporulation, as potential partners. These findings establish BA PrkA as a regulatory link between stress adaptation and sporulation in *B. anthracis*.

## INTRODUCTION

The life cycle of *Bacillus* species consists of three phases: vegetative growth, sporulation, and germination. During the vegetative phase, the bacteria grow and divide actively (1). When growth conditions become unfavorable, particularly due to nutrient limitation or environmental stress, the bacteria initiate sporulation, forming highly resistant endospores. These endospores are metabolically dormant, allowing the bacteria to survive under adverse conditions. Once conditions are favorable, the endospores undergo germination and re-enter the vegetative phase to resume growth and division.

Protein quality control and homeostasis are essential during these transitions between vegetative bacteria and spores, with ATP-dependent AAA+ (ATPases Associated with Diverse Cellular Activities) proteins playing key roles (2, 3). In *Bacillus subtilis* and *Bacillus anthracis*, members of the Clp, Lon, and FtsH families represent well-characterized AAA+ proteins involved in various cellular processes, including stress responses and sporulation (4–10). Given the key roles undertaken by AAA+ proteins in *Bacillus* spp., studying novel AAA+ domain-containing proteins could reveal new insights into their physiological roles in *B. anthracis*.

Here, we show that the *B. anthracis* protein BAS0518 (BA PrkA, now onward) is a homolog of *B. subtilis* PrkA (BS PrkA) with a predicted AAA+ ATPase domain that exhibits negligible protease activity *in vitro*. This contrasts with BS PrkA, which was reported to have ATP-dependent protease activity *in vitro*, although the activity was quite limited (11). Using a BA *prkA* deletion mutant, we demonstrate that BA PrkA is not required for normal growth during the vegetative phase. However, under osmotic stress conditions, the survival of the BA *prkA* deletion mutant is severely compromised. We further reveal that BA PrkA is not produced in actively growing vegetative bacteria when grown in a sporulation medium and begins to appear only at Stage II of sporulation. Additionally, BA *prkA* deletion results in downregulation of several sporulation-related genes at the RNA level, including *cotE* and *sigK* in the log phase, *sigK* and *spoIID* in the late exponential/early stationary phase, and *gerE* in the late stationary phase of bacterial growth.

We found that loss of BA PrkA results in the formation of low-viability, heat-sensitive spores with altered dye permeability, suggesting structural or functional defects in the spore. However, the specific nature of these defects remains to be determined. Lastly, through a BA PrkA interactome analysis, we identified potential BA PrkA interacting partners, ProA and EzrA, the alteration of which may contribute to the defects in stress response and sporulation, respectively. Together, these findings suggest that BA PrkA is required for efficient sporulation and stress response.

## RESULTS

### BA PrkA, a sequence ortholog of BS PrkA, has no detectable enzymatic activity

**Fig 1:**
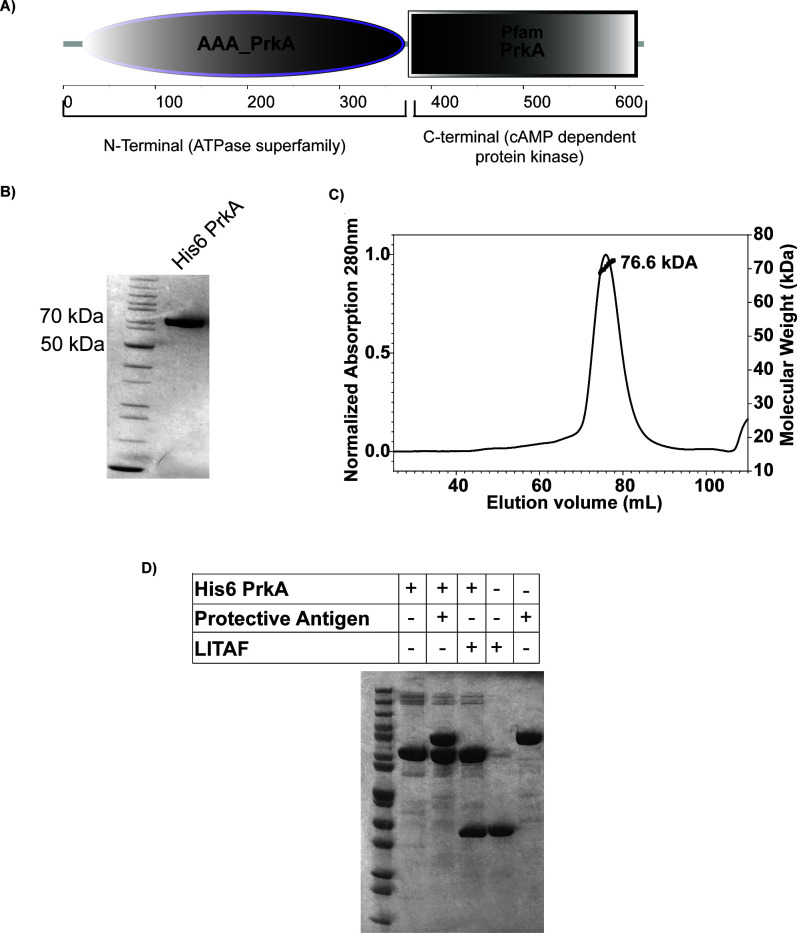
BA PrkA has no detectable protease activity. (**A**) Domain analysis of BA PrkA reveals that the N-terminal region of the BA PrkA protein belongs to the AAA+ superfamily of ATPases, while the C-terminal region is homologous to cAMP-dependent kinases. (**B**) Coomassie-stained SDS-PAGE showing the purified His6 PrkA protein. (**C**) Confirmation of purified His6 PrkA protein by size-exclusion chromatography coupled with multi-angle light scattering (SEC-MALS). The elution profile shows His6 PrkA at a molecular mass of 76.6 kDa, using buffer containing 5 mM CHAPS and 1 mM TCEP. (**D**) Coomassie-stained SDS-PAGE for measuring (non)proteolysis of LITAF and PA by monomeric His6 PrkA at 24 h, incubated at 37°C. His6 PrkA and substrates (LITAF, PA) were incubated separately at 37°C as controls. Enzyme and substrates were used at an amount of 2 µg per lane.

BA PrkA from *B. anthracis* (locus WP_268553006.1) is annotated as a serine/threonine protein kinase (PrkA family) based on sequence similarity to BS PrkA, a putative kinase previously identified through its distant homology to eukaryotic kinases and *in vitro* phosphorylation of a 60 kDa protein (12). Domain analysis of BA PrkA protein revealed the presence of two domains, an N-terminal domain related to the AAA+ (ATPases associated with diverse cellular activities) superfamily, and a C-terminal domain having distant homology to cAMP-dependent protein kinases (Fig. 1A).

Figure S1A describes the detailed domain analysis of BA PrkA. In the N-terminal part of the protein sequence, we found conserved Walker A and Walker B motifs, which are characteristic of ATPases known for ATP binding and hydrolysis (13). Other conserved motifs include sensor motifs for interaction with gamma-phosphate of bound ATP. The C-terminal part of the protein contains 11 conserved motifs typical of the Hanks-type kinases (14). Interestingly, the P-loop, which is important for ATP binding in Hanks-type kinases, is absent in the C-terminal part of BA PrkA. This may explain why purified BA PrkA exhibited no detectable autophosphorylation activity as reported previously for the homologous BS PrkA (Fig. S1A through C). However, BA PrkA did undergo low-level phosphorylation by the catalytic domain of another well-characterized *B. anthracis* Ser/Thr/Tyr kinase (STPK), PrkC (PrkC^cat^-GST) (15) (Fig. S1B and C).

To understand the nature of BA PrkA, first, we modeled its monomeric structure using AlphaFold 3 (16). Later, we classified BA PrkA as part of Clade III of the AAA+ superfamily based on this modeled structure. Clade III of the AAA+ superfamily of proteins is characterized by a pore loop 1 inserted between β-sheet 2 (β2) and β-sheet 3 (β3) of the core AAA+ domain (17). This feature was identified in the predicted monomeric structure of BA PrkA (Fig. S2A and B). Since most of the Clade III members form homo-hexameric complexes, such as YME1 (18), we modeled the homo-hexameric structure of BA PrkA using AlphaFold 3. The predicted structure shows the presence of a conserved aromatic-hydrophobic residue pair (228Y-229G) in pore loop 1 of BA PrkA, similar to what is observed in the representative Clade III structure of YME1 (PDB: 6AZ0) (18) (Fig. S2C and D).

A previous study on BS PrkA in *B. subtilis* suggested weak sequence similarity to the Lon-proteases and detected protease activity using α-casein as the substrate (11). This led us to investigate the protease activity of the *B. anthracis* BA PrkA. BAS0518 (BA *prkA*) was cloned into *Escherichia coli* expression vector pPro-Ex-Htc, which adds a hexa-histidine tag (His6) at the N-terminus of the protein. The protein was overexpressed and purified from the *E. coli* BL21(DE3) strain using Ni-NTA affinity chromatography, resulting in a single band of 76.6 kDa on SDS-PAGE (Fig. 1B). MS/MS analysis of this initial BA PrkA preparation showed it to contain a small amount (<0.1%) of an amidohydrolase (locus tag #ECBD_2279) of *E. coli*. This hydrolase may explain the weak protease activity we observed using a fluorescent assay with BODIPY FL-casein as the substrate, the same substrate used in the study of BS PrkA (Fig. S3A) (11). To circumvent this issue, further purification was performed using buffers containing 5 mM CHAPS along with 1 mM TCEP, which yielded a stable monomeric form of the protein, as determined by size-exclusion chromatography coupled with multiple angle light scattering (SEC-MALS) (Fig. 1C). To confirm the identity of the purified protein, we performed intact-mass analysis using mass spectrometry, which revealed a single peak of 76,639 Da, consistent with the calculated mass of the His6 PrkA protein. This confirmed the efficacy of our protein purification, and this protein was used for further biochemical characterization.

To assess the protease activity of the fully purified His6 PrkA, we used lipopolysaccharide-induced TNF factor (LITAF) and anthrax protective antigen (PA) as substrates. The selection of LITAF and PA was based on observations that they contain long, unfolded sequences (19, 20), which make them sensitive protease substrates. As shown in Fig. S3B, using SDS-PAGE, we observed a weak protease activity of the initial preparation of His6 PrkA (containing the putative amidohydrolase). However, the pure monomeric form of His6 PrkA did not cleave these substrates even after 24 h of incubation (Fig. 1D). Nonetheless, both His6 PrkA preparations (the initial sample and the purified form) produced the expected oligomeric species upon addition of ATP to the protease assay reaction (Fig. S3C) (18). Based on the AlphaFold 3 prediction (Fig. S2C and D) and the protease activity assay (Fig. 1D), we suggest that BA PrkA is structurally similar to the Clade III of AAA+ superfamily of proteins like YME1 protease of *Saccharomyces cerevisiae* (PDB-6AZ0) but lacks the active site residues necessary to perform protease activity.

### Physiological role of BA PrkA in *B. anthracis* stress responses

**Fig 2:**
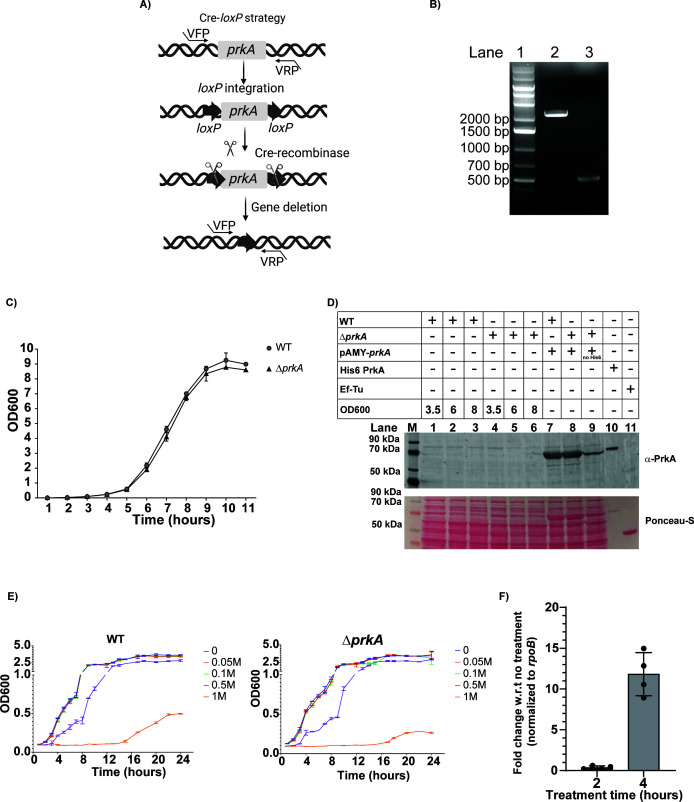
Deletion of BA PrkA does not affect growth under normal conditions but causes growth defects under ionic-osmotic stress. (**A**) Schematic diagram representing Cre-*loxP* based homologous recombination strategy for BA *prkA* gene deletion. VFP and VRP are BA *prkA* gene flanking primers. (**B**) Agarose gel showing PCR confirmation of BA *prkA* gene deletion. The deletion is verified by the presence of a 2,183 bp (BA WT) and 495 bp (BA *ΔprkA*) PCR amplicon generated using primers flanking the BA *prkA* gene (lane 2, 3). Lane 1: 1 kb DNA ladder. (**C**) Growth curves of BA wild-type (WT) (gray circle) and BA *ΔprkA* (black triangle) strains in LB medium. Values represent the mean optical density at 600 nm (OD_600_) ± standard deviation (SD) from *n* = 3 replicates. (**D**) Immunoblot with anti-PrkA to show temporal expression of BA PrkA protein at various time points during vegetative growth in LB medium. BA PrkA expression was analyzed using 40 µg of whole-cell lysate from BA WT and BA *ΔprkA* bacterial cultures collected at different OD_600_ values, as indicated above each lane. The total protein loaded in each lane was visualized by Ponceau staining. Lane M: protein ladder. Lane 7, 8, and 9: Overexpression strain of PrkA. Lane 10: 50 ng of purified His6 PrkA protein (positive control). Lane 11: Purified Ef-Tu protein (negative control). Data are representative of *N* = 3 independent experiments. Protein in each lane is normalized to total protein concentration estimated using the BCA method. (**E**) Growth curves of BA WT and BA *ΔprkA* strains in LB medium supplemented with varied concentrations of NaCl (0 M, 0.05 M, 0.1 M, 0.5 M, and 1 M). Y-axis values represent the mean optical density at 600 nm (OD_600_) ± standard deviation (SD) split in two segments (bottom OD_600_ = 0–1, and top OD_600_ = 2–5) from *n* = 2 experiments. (**F**) Bar graph showing the mean ± SD of BA *prkA* gene expression fold change following treatment with medium containing 1 M NaCl for the indicated time points, relative to cells grown in regular medium. Expression levels were normalized to *rpoB*. *N* = 4 independent experiments. Asterisks indicate statistical significance calculated using a two-tailed Student’s *t*-test (*P < 0.05, **P < 0.01, ***P < 0.005, ****P < 0.001)

Since *B. anthracis* survives in diverse and harsh environments, we examined the role of BA PrkA under different stresses, including ionic-osmotic stress, non-ionic osmotic stress, and oxidative stress. The AAA+ superfamily of proteins is known to play a central role in stress adaptation mechanisms across species (21). To investigate the physiological function of BA PrkA in *B. anthracis*, we generated an unmarked *prkA* gene deletion mutant (BA *ΔprkA*) using previously described methods (Fig. 2A and B) (22).

The BA *ΔprkA* strain was first assessed for its ability to grow under nutrient-rich conditions. In Luria–Bertani (LB) medium, BA *ΔprkA* exhibited a growth profile similar to the wild-type (BA WT) strain over ~10 h (Fig. 2C), indicating that BA PrkA is not required for growth under normal conditions (21). To further explore the physiological role of BA PrkA, we examined its expression during vegetative growth in LB medium at both the protein and transcript levels. Protein samples collected at OD_600_ values of 3.5, 6, and 8 (representing early-, mid-, and late-log phases) showed no detectable BA PrkA protein in actively growing cells (Fig. 2D). Transcript analysis similarly showed no detectable BA *prkA* expression during early and mid-log phases; however, BA *prkA* transcripts became detectable at the late-log phase (OD₆₀₀ = 8) (Fig. S3D).

Next, growth assays were used to examine the role of BA PrkA under different stress conditions, including ionic-osmotic stress (NaCl), non-ionic osmotic stress (20% sucrose), and oxidative stress (10 mM H₂O₂). Growth was monitored by OD_600_ measurements. As shown in Fig. 2E, BA *ΔprkA* exhibits a significant growth defect under 1 M NaCl stress, particularly after 16 h of incubation. Because BA PrkA is expressed in the late-log phase and provides protection to cells at a later stage of growth, we also performed endpoint assays using resazurin after 16 h of growth. Under ionic-osmotic stress, a significant decrease in fluorescence was observed only in 1 M NaCl condition, while no differences were detected under non-ionic (20% sucrose) or oxidative (10 mM H₂O₂) stress conditions (Fig. S4A through C). To validate the effect of 1 M NaCl, colony-forming units (CFU/mL) revealed approximately a 2.1-fold reduction in viability in BA *ΔprkA* relative to BA WT (Fig. S4D).

Given the sensitivity of BA *ΔprkA* to ionic-osmotic stress, we next examined whether BA *prkA* transcription itself is induced under such conditions. Indeed, BA *prkA* transcript levels increased by approximately 13-fold after 4 h of exposure to 1 M NaCl (Fig. 2F), supporting a role for BA PrkA in mediating osmotic stress resistance in *B. anthracis*.

### BA PrkA is abundantly expressed during sporulation and is required for spore viability

As shown earlier, PrkA plays a significant role in sporulation in *B. subtilis* (11, 23). Therefore, we assessed the involvement of BA PrkA in the sporulation process in *B. anthracis*.

**Fig 3:**
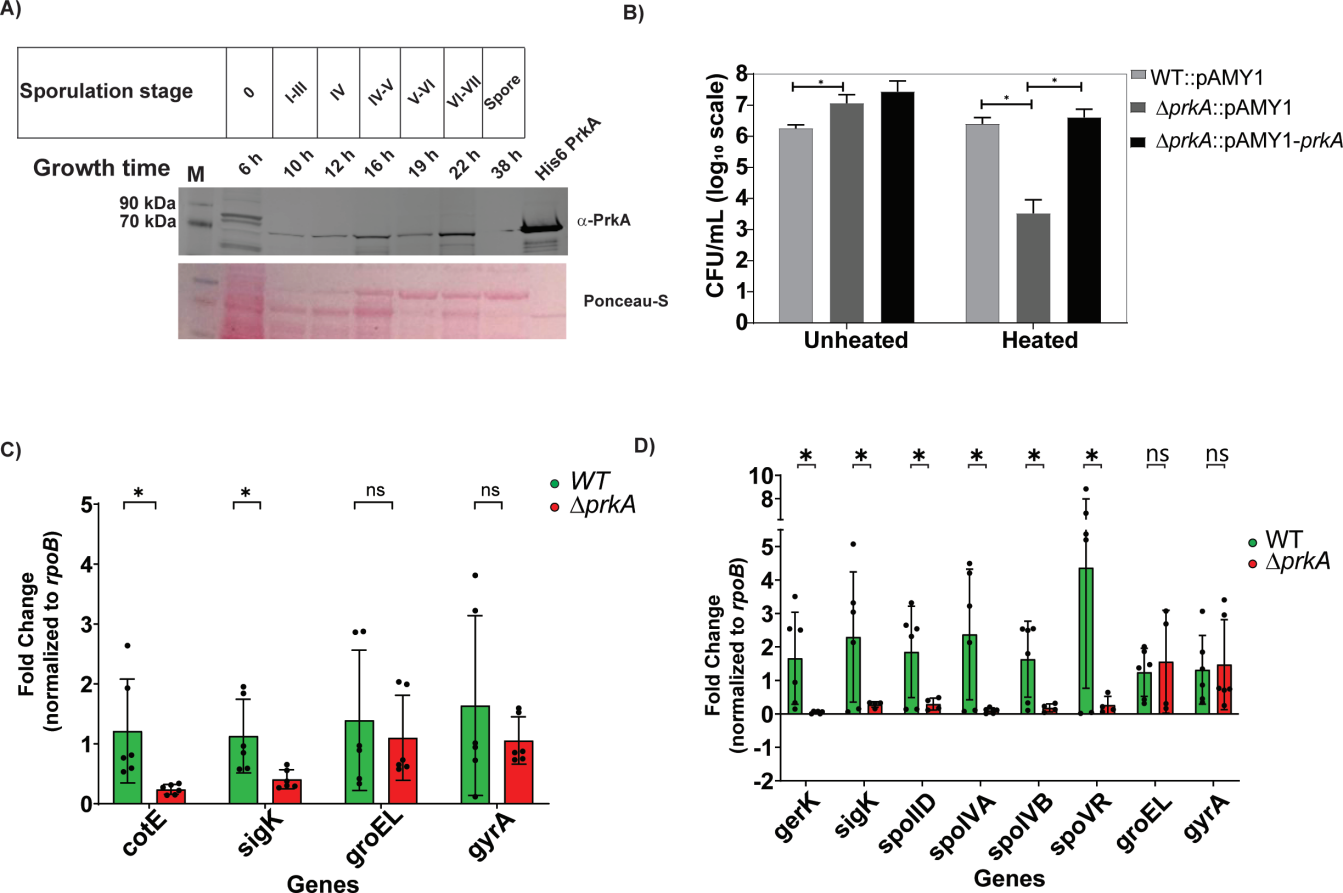
BA PrkA is highly expressed during sporulation stages, and its deletion reduces spore viability and alters sporulation gene expression. (**A**) Immunoblot with anti-PrkA to show temporal expression of BA PrkA protein at various time points corresponding to different stages of sporulation. Growth time and corresponding sporulation stages are mentioned on the top. Lane M: protein ladder. Lane 8: purified His6 PrkA protein (positive control). Data are representative of *N* = 2 experiments. Protein in each lane is normalized to total protein concentration estimated using BCA method. (**B**) Bar graph depicting mean ± SD of sporulation efficiency as colony-forming units per milliliter (CFU/mL) for BA WT::pAMY1, BA *ΔprkA*::pAMY1, and BA *ΔprkA*::pAMY1-*prkA* (complemented) strains. Values represent the mean CFU/mL ± standard deviation (SD) from *N* = 5 experiments. (**C, D**) RT-qPCR analysis represented as bar graphs showing mean ± standard deviation (SD) of relative expression of sporulation genes during early (C, Stage 0–III, growth time 8 h) and late (D, Stage V–VI, growth time 19 h) stages of sporulation. The expression levels were normalized to *rpoB* (housekeeping gene). *N* = 3 experiments. Green bars represent the wild-type (BA WT) strain, and red bars represent the BA *ΔprkA* mutant. Individual data points are shown as filled circles for both strains. Asterisks indicate statistical significance calculated using a two-tailed Student’s *t*-test (*P < 0.05, ns = non significant).

To begin, we evaluated BA PrkA expression at various stages of sporulation (Stages 0 to VII) (24) in bacteria cultured in sporulation medium. BA PrkA expression was subtly present in sporulating cells at Stage 0 and began to gradually increase until Stage VII, coinciding with the formation of the polar septum. BA PrkA protein levels decreased at later stages as the spores matured (38 h) (Fig. 3A; Fig. S5A and B), suggesting a role for BA PrkA in facilitating the successful completion of sporulation in *B. anthracis*.

Given this stage-specific expression of BA PrkA during sporulation, we next examined its effect on sporulation efficiency. As a control, a complemented strain of BA *ΔprkA* was created by ectopically expressing BA PrkA under an IPTG-inducible promoter in the pAMY1 plasmid (Table 1). Sporulation efficiency was measured by quantifying spore counts in BA WT::pAMY1, BA *ΔprkA*::pAMY1, and BA *ΔprkA*::pAMY1-*prkA* (complemented) strains under both unheated and heated (75°C) conditions. The BA WT strain produced approximately 6 × 10⁶ CFU/mL of mature (heat-resistant) spores after 5 days of nutrient starvation (Fig. 3B). However, the BA *ΔprkA* strain showed a significant 3-log reduction in mature (heat-resistant) spores. This sporulation defect was rescued in the complemented strain, confirming a major role of BA PrkA in sporulation. Similar observations were reported for *B. subtilis ΔprkA* mutants, which also showed reduced sporulation efficiency (11).

**TABLE 1:** Plasmids and strains used in this study

Name	Description/genotype	Resistance marker(s)	Source
pPro-Ex-HTc	*E. coli* expression vector with N-terminal His6 tag	Ampicillin	Invitrogen
pPro-Ex-HTc -*prkA*	pPro-Ex-HTc expressing BA PrkA in *E. coli*	Ampicillin	This study
pGEX5x3-PrkC^cat^-GST	Plasmid for expression of PrkC^cat^-GST in *E. coli*	Ampicillin	(15)
pAMY1	*B. anthracis-E. coli shuttle plasmid*; AmpR in *E. coli*; KanR in *B. anthracis*: IPTG-inducible promoter in *B. anthracis*	Ampicillin, kanamycin	(25)
pAMY1-*prkA*	pAMY1 expressing BA PrkA under IPTG promoter in *B. anthracis*	Ampicillin, kanamycin	This study
pAMY1-*prkA*-TEV-His6	pAMY1 expressing BA PrkA with TEV-cleavable His6 tag	Ampicillin, kanamycin	This study
*E. coli* DH5α	*E. coli F– endA1 glnV44 thi-1 recA1 relA1 gyrA96 deoR nupGpurB20 φ80dlacZΔM15Δ(lacZYA-argF)U169,hsdR17(rK–mK+), λ–*	–^*a*^	Invitrogen
*E. coli* BL21(DE3)	*E. coli* B strain: *F– ompT gal dcm lonhsdSB(rB–mB–)λ(DE3 [lacI lacUV5-T7p07 ind1 sam7 nin5]) [malB+]K-12(λS*)	–	Invitrogen
*E. coli* SCS110	*E. coli* SCS110 is an*endA–* derivative of the *JM110* strain *rpsL (Strr) thr leu endA thi-1 lacYgalK gal Tara tonAtsx dam dcm supE44 Δ(lac-proAB) [F’traAD36 proAB lacIqZ ΔM15]*	–	Stratagene
*B. anthracis* Sterne 34F2 (BA WT)	*B. anthracis* strain pXO1+, pXO2−	–	NIH, NIAID
BA *ΔprkA*	Null mutant *prkA* strain in *Bacillus anthracis* Sterne 34F2	–	This study
BA *ΔprkA*::pAMY1*-prkA-*TEV-His6	pAMY1*-prkA*-TEV-His6 in BA *ΔprkA*	Kanamycin	This study
BA WT::pAMY1	pAMY1 (empty vector) in BA WT	Kanamycin	This study
BA *ΔprkA::pAMY1*	pAMY1 (empty vector) in BA *ΔprkA*	Kanamycin	This study
BA *ΔprkA*::pAMY1*-prkA*	pAMY1*-prkA* in BA *ΔprkA*	Kanamycin	This study

^*a*^ “–”, not applicable.

The sporulation pathway is well-characterized in *Bacillus* species, where sigma factors *sigF*, *sigG*, *sigE*, and *sigK* regulate sporulation-related genes in a stage-specific manner (26). For example, *sigF*, expressed in the forespore, regulates *spoIVB*, while *sigE*, expressed in the mother cell, regulates *spoVB* and *spoIID*. Similarly, *sigG* (forespore) controls *gerD*, and *sigK* (mother cell) regulates *gerE*. Therefore, we analyzed the expression of key sporulation-related genes across early and late stages of sporulation (26). At the early stages of sporulation (Stages 0–III), we observed significant reductions in the expression of *cotE* (4-fold) and *sigK* (2.5-fold) in the BA Δ*prkA* strain (Fig. 3C; Fig. S5C). At Stages V–VI of sporulation, other sporulation genes, such as *spoIID*, *spoIVA*, *spoIVB*, *spoVR*, and *gerK*, also exhibited reduced expression, suggesting a pleiotropic effect of BA PrkA in regulating sporulation (Fig. 3D; Fig. S5D). Based on these gene expression analyses, we suggest that the BA *ΔprkA* mutant produces a strong defect that acts relatively early and then throughout the sporulation process, affecting expression of all the sporulation genes examined (23). This dysregulation of sporulation genes provides a mechanistic basis for the heat-sensitive phenotype observed in BA *ΔprkA* spores.

### BA PrkA is required for efficient sporulation

**Fig 4:**
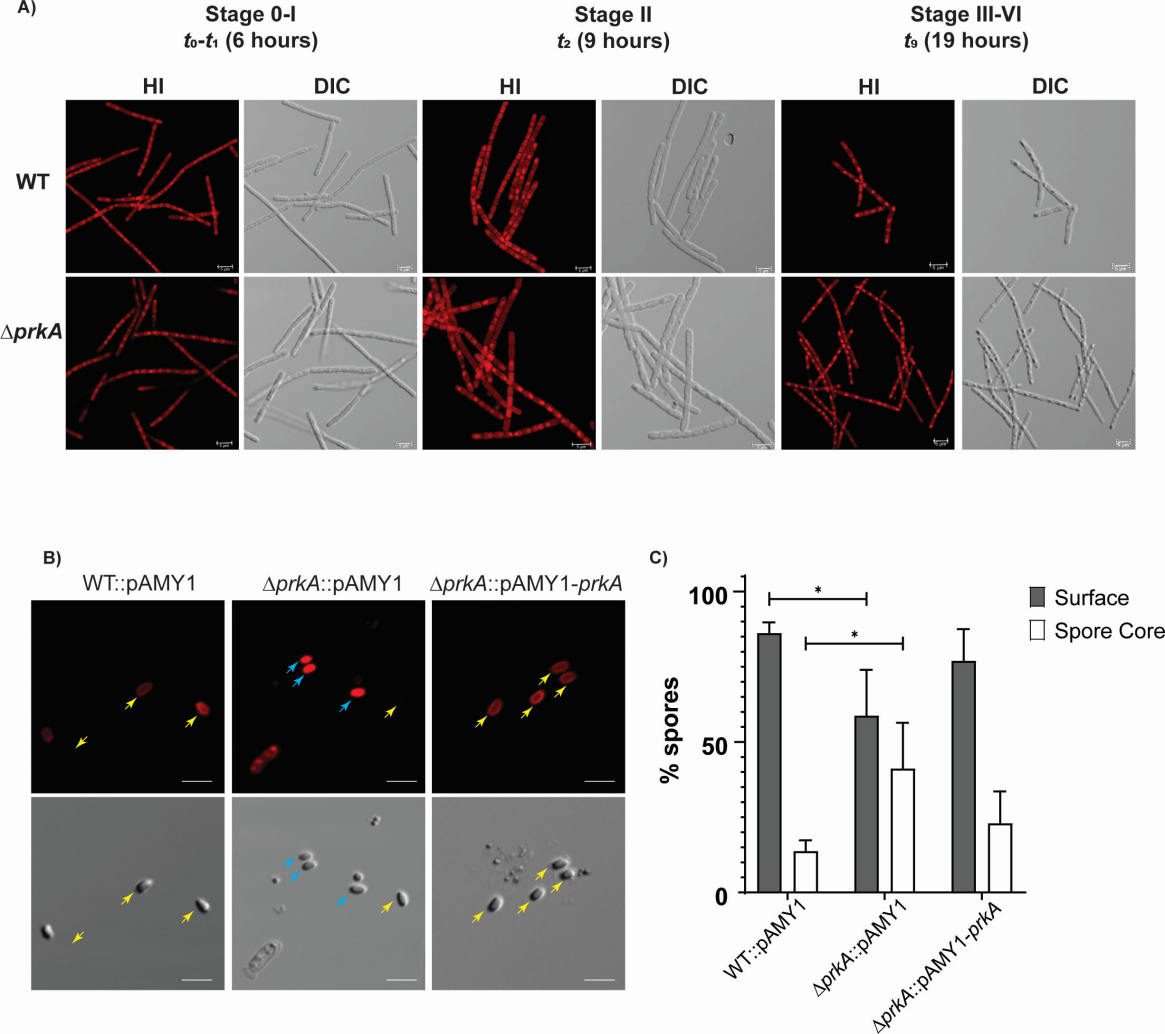
Deletion of BA *prkA* affects mature spore formation: (**A**) Representative hexidium iodide (HI) stained and differential interference contrast (DIC) images of BA WT and BA *ΔprkA* at different sporulation stages (6 h [*T*_0_*–T_1_*], 9 h [*T_2_*], and 19 h [*T*_9_] time intervals); Stages 0 to VI. Scale bar (2µm) is depicted in all images. *N* = 3 biological replicates. (**B**) Representative HI stained and DIC images of BA WT, BA *ΔprkA*, and BA *ΔprkA*::pAMY1-*prkA* spores. Scale bar = 2 µm. Yellow arrows indicate spores with non-permeabilized weak surface staining (hexidium iodide), and blue arrows indicate spores with strong, diffused, permeabilized hexidium iodide staining of spore core. Data are from *N* = 3 biological replicates. (**C**) Bar graph showing mean ± SD of the percentage of spores exhibiting spore core staining versus surface-localized staining. Data are from >2,000 spores counted across three biological replicates. Asterisks indicate statistical significance calculated using a two-tailed Student’s *t*-test (*P < 0.05).

To understand why BA *ΔprkA* shows defects in sporulation, we used hexidium iodide staining to track the progression of sporulation and the generation of mature spores in this strain. Hexidium iodide stains DNA and shows its cellular distribution during different stages of sporulation. Fluorescence microscopy using hexidium iodide showed that BA *ΔprkA* cells underwent the same sporulation stages as the BA WT and formed spores (Fig. 4A). Later, we investigated whether the loss of BA PrkA produced structural defects in the spores, given the significant reduction in the expression of sporulation-related genes (*cotE*, *spoIVA*, and *spoIVB*). The integrity of the spore integuments (the multilayered protective layers including spore membranes, spore cortex, spore coats, and exosporium) was tested using spore-impermeable hexidium iodide on the BA *ΔprkA* spores. As shown in Fig. 4B and C, the unheated spores from the BA *ΔprkA* strain show significant spore core staining of hexidium iodide. This phenotype was not present in the WT and the complemented (BA *ΔprkA*::pAMY1-*prkA*) strains, suggesting a weak and permeable spore integuments formation in the BA *ΔprkA* strain. Therefore, we propose that the weak spore integuments result in the development of the heat-sensitive phenotype in BA *ΔprkA* spores.

### BA PrkA interactome consists of proteins involved in septum formation, stress response, and metabolism

**Fig 5:**
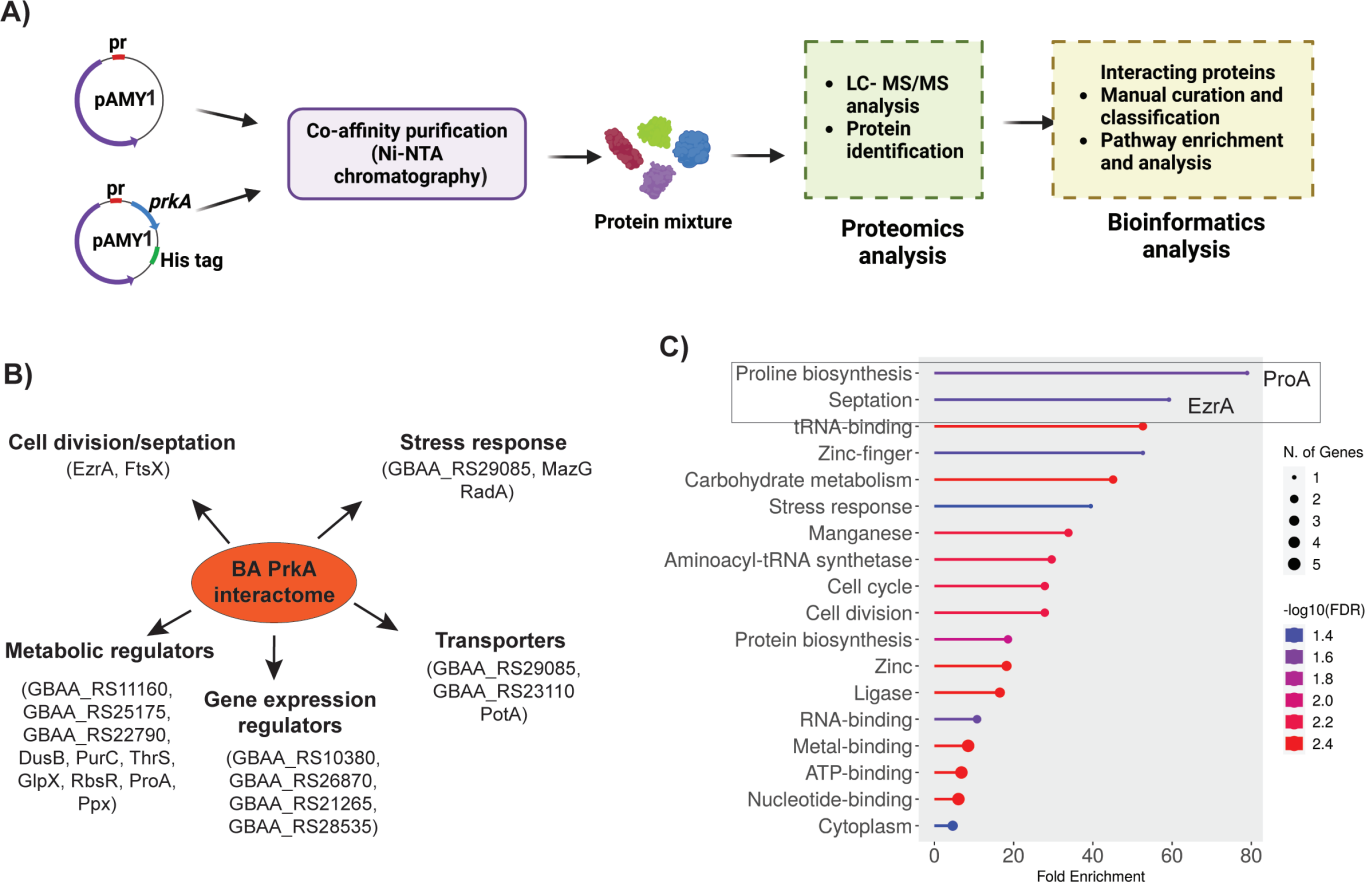
BA PrkA interactome analysis reveals binding partners potentially linked to compromised spore viability and osmotic stress sensitivity. (**A**) Schematic representation of the co-affinity purification strategy used to isolate the BA PrkA interactome, followed by mass spectrometry analysis and subsequent interactome characterization. (**B**) Broad classification of the 22 proteins identified as BA PrkA interacting partners. A detailed table listing these proteins is provided in the Tables S1 and S2. (**C**) Pathway enrichment analysis showing statistically enriched pathways in the BA PrkA interactome. Notably, EzrA and ProA are highlighted, as they are associated with sporulation and osmotic stress.

To uncover the mechanism by which BA PrkA influences sporulation in *B. anthracis*, we identified proteins that interact with BA PrkA (i.e., “interactors”). As described in the “Materials and Methods” section, co-affinity purification followed by mass spectrometric analysis was performed using whole-cell lysates from *B. anthracis* cells expressing His6 PrkA under an IPTG-inducible promoter. The cells were harvested at the early logarithmic growth phase (OD₆₀₀ ≈ 2.0) (Fig. 2C), a stage selected to capture BA PrkA-associated proteins present before major sporulation-specific cellular changes occur. This approach was based on our observation that BA PrkA expression begins to increase toward the end of vegetative growth and the onset of sporulation (Fig. 3A). A total of 22 co-purifying proteins were identified as potential direct or indirect interactors of BA PrkA (workflow shown in Fig. 5A; complete list in the Tables S1 and S2). These interactors were grouped into five major functional categories—cell division/septation, metabolic processes, stress response, gene regulation, and transport (Fig. 5B).

Pathway enrichment analysis performed using ShinyGO (27) mapped 11 of the 22 proteins to one or more functional categories (Fig. 5C). Among these, ProA and EzrA emerged as the most enriched proteins. ProA, a critical enzyme in proline biosynthesis, has been previously linked to osmotic stress tolerance (28, 29). EzrA, a key regulator of septation ring formation, plays a crucial role in the sporulation process (30). Additionally, Ppx, another identified interactor, is implicated in both osmotic stress response and sporulation efficiency (31). These findings suggest that BA PrkA exerts its effect on sporulation and stress responses by interacting with proteins such as ProA, EzrA, and Ppx. These findings highlight the central role of BA PrkA in coordinating stress responses, metabolic adaptation, and cellular processes essential for spore maturation and viability.

## DISCUSSION

In this study, we characterized a novel *B. anthracis* protein, BAS0518 (BA PrkA), and uncovered its functional role in sporulation and stress adaptation. BA PrkA contains a putative N-terminal AAA+ ATPase domain and a C-terminal kinase domain, with a strong homology (88% identical at protein level) to BS PrkA. Our findings highlight that BA PrkA is a canonical Clade III AAA+ protein and plays a regulatory role in the cellular processes of *B. anthracis*.

No kinase activity was detected in the purified BA PrkA, likely due to the lack of a P-loop in its kinase domain (Fig. S1A through C), as reported previously for the homologous BS PrkA (14). Furthermore, as shown in Fig. 1A through D, purified His6 PrkA displays negligible protease activity against substrates like LITAF and PA, even after incubation for 24 h. Size-exclusion chromatography coupled with multi-angle light scattering (SEC-MALS) (32) revealed a monomeric form of purified His6 PrkA. Notably, earlier preparation done in buffers without CHAPS and TCEP contained trace amounts of an amidohydrolase contaminant (<0.1%) and had very weak protease activity. Similar issues may explain previously reported low-level protease activity in BS PrkA (Fig. S3) (11). Upon re-purification under reducing conditions, BA PrkA had no detectable protease activity. These findings highlight the need for stringent purification to avoid misleading enzymatic activity and suggest that BA PrkA may exert its regulatory effects through non-enzymatic mechanisms.

Functional analyses revealed that deletion of *prkA* in *B. anthracis* compromises growth under osmotic stress and alters the transcription of several key sporulation-related genes, ultimately leading to defects in mature heat-resistant spore formation (Fig. 2 to 4). Although the BA *ΔprkA* strain progresses through the morphological stages of sporulation similarly to the BA WT strain, its spores display structural and functional abnormalities, resulting in increased heat sensitivity. Gene expression analysis revealed significant downregulation of *cotE* and *gerK*, which are critical for spore-coat formation and spore germination (33, 34), respectively. Transcriptional analysis of several genes regulated by sporulation-related sigma factors*—sigF*, *sigG*, *sigE*, and *sigK*—confirms that BA PrkA exerts pleiotropic effects during sporulation. The downregulation of other essential sporulation genes, such as *spoIID*, *spoVR*, and *gerK*, in the BA *ΔprkA* mutant also suggests that BA PrkA has a broad effect on the sporulation pathway. The altered hexidium iodide dye permeability observed in BA *ΔprkA* spores, together with the downregulation of multiple sporulation genes, suggests that the weakened spore integuments may arise from combined defects in spore-coat organization, core dehydration, and/or inner membrane integrity.

To elucidate the mechanistic basis of BA PrkA’s role in the *B. anthracis* life cycle, we mapped its interactome through co-affinity purification and mass spectrometry (Fig. 5). The proteins identified as potential interacting partners (“interactors”) of BA PrkA are involved in septum formation, metabolic pathways, stress responses, and gene regulation. Notably, ProA, EzrA, and Ppx emerged as key interactors linked to the observed phenotypes. ProA, a central enzyme in proline biosynthesis, supports osmotic stress adaptation by maintaining osmotic balance (28, 29). EzrA, a septation ring regulator, is essential for cell division and may have a novel role in spore maturation and viability (30). Ppx, implicated in stress responses and sporulation efficiency (31), further connects BA PrkA to stress adaptation mechanisms.

The absence of proteolytic activity in BA PrkA suggests an alternative mechanism of action, with BA PrkA acting as a competitive inhibitor to other AAA+ proteins or as a molecular scaffold. Such proteins are known to regulate processes by binding substrates or cofactors without catalysis (35, 36), and this paradigm may extend to BA PrkA. By interacting with key players, such as ProA, EzrA, and Ppx, BA PrkA could serve as a regulatory hub, modulating sporulation and stress response pathways in *B. anthracis*.

### Conclusion

In summary, this study suggests that BA PrkA may act as a competitive inhibitor to other AAA+ proteins or serve as a molecular scaffold in *B. anthracis*, playing crucial roles in sporulation and stress adaptability. These findings provide new insights into the molecular mechanisms of spore development and stress response in *B. anthracis*, underscoring the importance of BA PrkA in these processes.

## MATERIALS AND METHODS

### Bacterial strains and growth conditions

*E. coli* strains DH5α and SCS110 were used for cloning, and BL21(DE3) was used for the expression of recombinant proteins (Table 1). Cultures were grown in Luria–Bertani (LB) broth (Difco) supplemented with appropriate antibiotics at 37°C with constant shaking at 200 rpm. Ampicillin and kanamycin were used at final concentrations of 100 µg/mL and 25 µg/mL, respectively.

*B. anthracis* Sterne 34F2 strain (BA WT) was used as the background strain for the construction of all other strains (Table 1). All *B. anthracis* strains were grown in LB broth (Difco) at 37°C with constant shaking at 200 rpm or in sporulation medium at 30°C with shaking at 200 rpm (8). Kanamycin was used at final concentrations of 15 µg/mL.

### Cloning, expression, and protein purification

The coding sequence of BAS0518 (BA *prkA*) was PCR-amplified using gene-specific forward and reverse primers (Table 2) containing BamHI and XhoI restriction enzyme sites at the 5′ and 3′ ends. The PCR product was cloned into the pPro-Ex-HTc vector using the BamHI and XhoI restriction enzymes (NEB), followed by ligation using T4 DNA ligase (NEB). The ligation mixture was transformed into *E. coli* DH5α, and clones were sequenced using Sanger sequencing (Psomagen).

**TABLE 2:** Primers used for cloning, gene deletion, and qPCR analysis^*a*^

Primer name	Primer sequence *5´→ 3´*
Primers for cloning and protein purification	
BAS0518 (BA *prkA*) FP (BamH1)	CCGGGATCCATATGGATATTCTAAAAAAAATT
BAS0518 (BA *prkA*) RP (Xho1)	CCGGCTCGAGCTATCGATTTAGCAGGCTACCTA
Primers for BA *prkA* knockout preparation	
SC LFP	ACGTCTCGAGCCCGATTGCAGTTAATATTTCAGCC
SC LRP	ACGTACTAGTCCTTCCCATTGTAAGCGTTCTTCT
SC RFP	ACGTCTCGAGGAGCACGGCTATAATTCTTCTTCAGC
SC RRP	ACGTACTAGTGGTCCTTTTTGCTTTTGACCACC
0518 VFP (internal primers)	TGATTTAAAGAATGTTTGAGGACATCCTAGCA
0518 VRP (internal primers)	CTTTGCAACATATTGTATTGTGTTGGCTTTTT
Real-time PCR primers	
*spoVR* RT FP	GCACTGCAATATGCGATAGCG
*spoVR* RT RP	CGAGTTCGTATATTTTGCTAAGACCTAAATCG
*spoIVA* RT FP	GGGCGCAGGATGAACTACC
*spoIVA* RT RP	GAGTATTAATCATGCGCGGGCC
*spoIVB* RT FP *spoIVB* RT RP	CCGCTTCGGACGTTTATTTCATC CCAGCAAGCTGAAACACCATATCTG
*spoIID* RT FP	GTGCTTCTCATAGCGCTCGTTATC
*spoIID* RT RP	CACATACTCCTCCATAGGTAATGATTCTACC
*sigK* RT FP	GAAGAACAATGCGTTTCCGCAG
*sigK* RT FP	GCCCGATTGTACCAATTGAAATTAAATCT
*gerE* RT FP	CCTTTACTCACAAAGAGAGAGAGAGAAG
*gerE* RT RP	CGAGCTCTCCCATACGAAGAAG
*cotE* RT FP	GCAGTGGTTGGAAAAGGACGTAAG
*cotE* RT RP	CTGTTACAACTTCTGTCTTTGTATTGCCATC
*gerD* RT FP	CCGATCAAGGTAAGAAAGCCATTCA
*gerD* RT RP	GCAAATTTCGATGAGAACTCAGGGTC
*dps2* RT FP	AACAAGTAGCAGACTGGAGCG
*dps2* RT RP	CTTTCATTGTTGCTACTGGTTTGCCG

^*a*^ The underlined sequences represent the respective endonuclease sites used for cloning.

Recombinant plasmids were subsequently introduced into *E. coli* BL21(DE3) cells for protein expression. A starter culture of the recombinant *E. coli* strain containing the desired plasmid was grown overnight in 5 mL of LB broth supplemented with ampicillin (100 μg/mL). This primary culture was then used to inoculate a 1 L secondary culture in LB broth with ampicillin (100 μg/mL), which was incubated at 37°C with shaking at 200 rpm. The culture was grown until the OD_600_ reached 0.6–0.8. At this point, protein expression was induced by adding IPTG to a final concentration of 1 mM, and the culture was incubated for an additional 3 h at 37°C. Cells were then harvested by centrifugation at 8,000 rpm for 5 min, and the resulting pellet was washed with 1× PBS.

The cell pellet was resuspended in sonication buffer (50 mM Tris-HCl [pH 8.5], 5 mM β-mercaptoethanol, 1× protease inhibitor cocktail [Roche Applied Science, USA], and 300 mM NaCl) and subjected to sonication (nine cycles at 20% amplitude, 10 s on, 30 s off). The lysate was then centrifuged at 13,000 rpm for 1 h. The supernatant containing the recombinant protein was purified by immobilized metal affinity chromatography (IMAC) and eluted with 200 mM imidazole. The purified proteins were pooled, dialyzed for downstream assays, and stored at −80°C.

For purification of the GST-tagged catalytic domain of PrkC (PrkC^cat^-GST), the recombinant protein was purified using the method described earlier (15).

### Generation of single gene knockout strains in *B. anthracis*

The BA *prkA* knockout strain in *B. anthracis* was generated using the method as described (22). Briefly, two single-crossover plasmids derived from the temperature-sensitive shuttle vector pSC were used sequentially to insert *loxP* sites flanking the regions of interest in the BAS0518 genes. A third temperature-sensitive plasmid, pCrePAS (22), expressing Cre recombinase, was then employed to excise the DNA region between the *loxP* sites, resulting in the deletion of the target gene from the *B. anthracis* Ames 35 genome. Positive colonies were confirmed by PCR using primers flanking the BA *prkA* gene (Table 2).

### Generation of complement strain of BA *prkA*

The coding sequence of BA *prkA* was synthesized as a gBlock (Integrated DNA Technologies) with ends homologous to the pAMY1 vector that was digested with HindIII/SalI. Using NEB HiFi, the gBlock was cloned into the digested pAMY1 to generate pAMY1-*prkA* (Table 1). The complemented strain (BA *ΔprkA*::pAMY1*-prkA*) was generated by electroporating the BA *ΔprkA* strain with pAMY1-*prkA* plasmid.

### Growth kinetics under LB medium

The primary cultures of BA WT and BA BA *ΔprkA* strains were grown in LB broth at 37°C and 200 rpm overnight. The overnight culture was added as the inoculum to begin secondary cultures in triplicates at starting OD_600_ of 0.05 in similar growth conditions in 50 mL of the LB medium in a 250 mL baffled flask and incubated at 37°C with shaking at 220 rpm. OD_600_ measurements were taken every hour using 1 cm cuvette on Ultrospec 3300 Pro (Amersham Biosciences) until the cells reached the stationary phase.

### PrkA phosphorylation assay

Autophosphorylation activity of His6 PrkA was assessed using an anti-phosphothreonine (anti-pThr) antibody, following a previously described method (37). Briefly, 500 ng of His6 PrkA was incubated with or without 1 mM ATP in a reaction buffer containing 20 mM HEPES (pH 7.4), 5 mM MgCl_2_, 5 mM MnCl_2_, and 1 mM DTT for 30 min at 25°C.

As a positive control for kinase activity, we used the GST-tagged catalytic domain of PrkC (PrkC^cat^-GST), along with purified elongation factor Tu (Ef-Tu), myelin basic protein (MBP), and GST as substrates (37). For each indicated lane, 500 ng of PrkC^cat^-GST was combined with 5 µg of Ef-Tu, 10 µg of MBP, or 5 µg of GST. Each reaction was incubated under the same conditions as described above. To test whether His6 PrkA could serve as a substrate for another kinase, His6 PrkA was also incubated with PrkC^cat^-GST under identical assay conditions.

Reactions were resolved on a 4–20% SDS-PAGE gel, and proteins were transferred onto a nitrocellulose membrane using wet transfer at 4°C for 2 h at 120 V. Nitrocellulose membranes were probed with rabbit anti-pThr (Thermo Fisher) and rat anti-PrkA (see below) primary antibodies, followed by anti-rabbit IgG (H&L) DyLight 700–conjugated antibody (Rockland) and anti-rat IgG (H&L) DyLight 800–conjugated antibody (Rockland). Imaging was performed using a LI-COR Odyssey CLx system, and band intensities were quantified using Image Studio software (LI-COR).

### Protease assay

Protease activity of His6 PrkA was assessed using two methods: (i) fluorescently labeled substrate (α-casein) and (ii) visual analysis on SDS-PAGE.

In the first method, protease activity was measured using the EnzChek Protease Assay Kit. BODIPY FL-casein served as the substrate, and reactions were set up in Corning 96-well solid black flat-bottom plates. Each reaction (100 µL) contained 1 µg of His6 PrkA. The protease activity was measured by adding 100 µL of a 10 µg/mL working solution of BODIPY casein. The plate was incubated at room temperature for 2–24 h, and fluorescence was measured at 485 nm excitation/535 nm emission.

For the second method, reactions were set up with 0.1 μg/μL of His6 PrkA and 0.1 μg/μL of substrates (α-casein, LITAF, and PA) in 1× digestion buffer (40 mM Tris/HCl, pH 7.5, 1 mM MgCl₂, and 1 mM ATP) and incubated at 37°C for 16 or 24 h. Controls were prepared with enzyme or substrate alone. Reactions were terminated by adding 5× SDS sample buffer and analyzed by 12.5% SDS-PAGE.

The oligomeric form of purified His6 PrkA was observed on Native PAGE (PhastSystem, Pharmacia). Two micrograms of each protein (initial preparation and pure form) were resuspended in 1× digestion buffer (with and without ATP) and resolved on 8% native gel on PhastSystem, followed by Coomassie staining.

### Size-exclusion chromatography coupled with multi-angle light scattering (SEC-MALS)

To assess the purity and molecular weight of His6 PrkA, protein was injected onto a HighLoad 16/60 Superdex 200 column connected to an AKTA Pure HPLC system. The column was operated in a buffer containing 200 mM Tris-HCl, pH 8.0, 300 mM NaCl, 5 mM CHAPS, 1 mM TCEP, and 10% glycerol. The HPLC system was connected to a DAWN HELEOS II detector featuring a quasi-elastic light scattering module, along with an Optilab T-rEX refractometer (Wyatt Technology). Data were analyzed using the ASTRA 8.1.2.1 software (Wyatt Technology Europe).

### Growth under stress conditions

Growth of BA WT and BA *ΔprkA* was assessed under various stress conditions, including ionic-osmotic stress (0, 0.05, 0.1, 0.5, and 1 M NaCl), non-ionic osmotic stress (0.5%, 5%, 10%, and 20% [wt/vol] sucrose), and oxidative stress (0, 0.1 mM, 1 mM, and 10 mM H₂O₂). Bacteria were inoculated at a starting OD_600_ of 0.5 into 5 mL of the respective media in 50-mL tubes and incubated at 37°C with shaking at 220 rpm. OD_600_ measurements were taken every hour using a 96-well microtiter plate on a Victor3 plate reader. Cell viability was assessed by plating serial dilutions on LB agar and incubating the plates at 37°C for 16 h to determine colony-forming units (CFU/mL). Endpoint assays were conducted following a 16-hour incubation period. Both strains were cultured overnight in LB medium at 37°C with shaking at 200 rpm. On the following day, the cultures were inoculated with starting OD_600_ of 0.05 into 200 µL of stress media in a 96-well microtiter plate with lid and incubated overnight at 37°C and 200 rpm in a humidity chamber to monitor growth. To evaluate cell viability at the assay endpoint, resazurin dye (R&D Systems) was added to each well at a 1:10 dilution. Fluorescence was measured using a Victor3 plate reader with excitation/emission settings of 540/590 nm.

### *B. anthracis* spore preparation and quantification

The BA WT::pAMY1, BA *ΔprkA*::pAMY1, and BA *ΔprkA*::pAMY1-*prkA* strains were grown overnight in LB broth at 37°C with shaking at 200 rpm. The overnight cultures were spread onto LB-agar plates containing 15 µg/mL kanamycin and 250 µM IPTG. Plates were incubated at 37°C for 24 h and then shifted to 30°C for 9 days to permit completion of sporulation by existing sporulating cells. Spore formation was monitored using DIC microscopy. Spores were harvested by resuspending them in 10 mL of deionized water, followed by washing three times at 12,000 × *g* for 15 min with sterile deionized water. The spores were then resuspended in 5 mL of sterile deionized water. Half of the spore preparation was heat-treated at 75°C for 30 min, while the remaining half was kept at room temperature in sterile deionized water. Spore viability was assessed by plating 100 µL of 10-fold serial dilutions of both heated and unheated spores on LB-agar plates containing 15 µg/mL kanamycin and 250 µM IPTG.

### PrkA antibody generation and temporal expression using immunoblotting

Anti-PrkA antibodies were produced in female Fischer (CDF) rats (Charles River) by priming with 50 µg of purified His6 PrkA (antigen), followed by two boosts with 25 µg of antigen at 2 weeks and 5 weeks post-priming. All immunizations were by subcutaneous route, and antigen was pre-mixed with an equal volume of Alhydrogel (Invivogen). Rats were bled 2 months post-priming and tested for anti-PrkA antibody by standard ELISA. All animal immunizations were done in accordance with protocols approved by the NIAID ACUC (animal protocol LPD8E).

For the temporal expression of BA PrkA, 5 mL cultures of bacteria in vegetative and sporulating stages (selected based on microscopic analysis) (24) were collected and lysed in 1 mL lysis buffer (6 M urea, 50 mM NH_4_CO_3_, 1 mM DTT) by performing seven cycles of bead beating with 0.1 mm zirconia beads (BioSpec) using Precellys Evolution homogenizer (Bertin Technologies, France) at 9,000 rpm, with 20 s ON and 1 min OFF. The lysates were centrifuged at 13,000 rpm for 30 min, and total protein concentration was estimated using the BCA method (Pierce, Invitrogen). Since BA PrkA expression was tested in different stages of growth and sporulation, lysate volumes were normalized to the total protein concentration, and 20 µg of total lysate from each condition was resolved on a 4–20% SDS-PAGE gel (Invitrogen). The proteins were transferred onto a nitrocellulose membrane using the iBlot2 (Invitrogen). Blots were stained with Ponceau S stain, imaged, and washed with 1× PBS using standard protocol to confirm the equal loading in each lane. The blot was blocked and probed using rat polyclonal anti-PrkA at a dilution of 1:5,000 in LI-COR blocking buffer. Rat IgG (H&L) DyLight 800–conjugated antibody (Rockland) was used as the secondary antibody for immunoblotting, and blots were scanned on the LI-COR Odyssey CLx.

### Fluorescent microscopy for spores and sporulation stages

Sporulation stages in BA WT and BA *ΔprkA* were monitored using the cell-permeable dye hexidium iodide at 100 µg/mL. Briefly, bacterial cells were synchronized by streaking single colonies of each strain onto LB-agar plates and incubating at 37°C for 16 h. Single colonies from each strain were then inoculated into 5 mL sporulation media (8 g LB broth/L supplemented with 85.5 mM NaCl, 0.025 mM ZnSO₄, 0.6 mM CaCl₂, 0.3 mM MnSO₄, 0.8 mM MgSO₄, and 0.02 mM CuSO₄, pH 5.0–5.5) and incubated at 30°C with shaking at 220 rpm to induce sporulation.

At different time points between 13 and 16 h post-inoculation, 200 µL of each culture was collected, washed with 1× PBS, and incubated with 100 µg/mL hexidium iodide for 20 min at room temperature. The cells were then fixed with 4% paraformaldehyde in 1× PBS overnight at 4°C. Fixed cells at different sporulation stages were imaged using a Leica SP8 microscope (690/730).

For the hexidium iodide (HI) staining assay in spores, WT::pAMY1, BA *ΔprkA*::pAMY1, and BA *ΔprkA*::pAMY1-*prkA* strains were used. Spores were prepared, stained, and imaged as described above. Spores exhibiting only surface staining were considered mature, heat-resistant spores, while those staining in the spore core were classified as heat-sensitive spores. The number of surface-stained and spore core-stained spores was manually counted.

### RNA extraction and quantitative real-time PCR

BA WT and BA *ΔprkA* strains were grown in triplicates to the different growth phases as mentioned below, and RNA was extracted using a modified hot lysis method as previously described (38–40). Briefly, cells harvested at the desired growth stage were resuspended in 500 µL TRIzol (Invitrogen). To this, 400 µL of hot lysis buffer (50 mM Tris, pH 8.0; 1% SDS; 1 mM EDTA) and 400 µL of DEPC-treated zirconia beads were added. The cell suspension was incubated at 65°C for 15 min with intermittent vortexing every 5 min. After cooling on ice, 100 µL of chloroform per mL of TRIzol was added, and the suspension was centrifuged at 9,500 × *g* for 15 min at 4°C to separate the RNA in the aqueous phase. RNA was precipitated by adding 0.5 M LiCl and three volumes of ice-cold isopropanol, followed by incubation at −80°C for 2 h. The RNA was pelleted by centrifugation at 16,000 × *g* for 20 min at 4°C, washed with 70% ethanol (Merck), and resuspended in nuclease-free water after air-drying. DNA contamination was removed by treating the sample with DNase (Ambion) following the manufacturer’s instructions. The purified RNA was then used for cDNA synthesis using a first-strand cDNA synthesis kit (Thermo Fisher).

To analyze the expression of sporulation genes at early (Stage 0–III, growth time 8 h, Fig. 3C; Fig. S5C) and late (Stage V–VI, growth time 19 h, Fig. 3D; Fig. S5D) stages of sporulation, cDNA was synthesized from the RNA samples and analyzed using gene-specific primers and SYBR Green master mix (Roche). Additionally, growth-dependent expression of the BA *prkA* gene was assessed with the cDNA synthesized from RNA isolated from BA WT and BA *ΔprkA* at OD_600_ values of 3.5, 6, and 8 in regular LB media, and Stage IV–V (growth time 16 h) in sporulation media. Similarly, to analyze the expression of BA *prkA* gene under ionic-osmotic stress, cDNA was synthesized from RNA isolated from WT strain first grown to OD 1 in regular LB media and then transferred to fresh LB media supplemented with 1 M NaCl for the time points indicated in Fig. 2F. Reactions (10 µL each) were run in triplicates with no-template controls in a Quant Studio 7 (ThermoFisher Scientific). The housekeeping gene *rpoB* (encoding DNA-directed RNA polymerase subunit beta) (41) was used for normalization. The primers used in this study were designed to amplify PCR products of 100–150 bp.

### Co-affinity purification and mass spectrometry

*B. anthracis* strains were cultured in 200 mL of LB broth. The strains used included BA WT::pAMY1 and BA WT::pAMY1-*prkA*-TEV-His6. Once cultures reached an OD_600_ of ~0.2, 50 µM IPTG was added, and growth continued until the cultures reached an OD_600_ of ~2. Cells were harvested by centrifugation at 6,200 × *g* for 10 min at 4°C, and the wet weights were recorded. Pellets were snap-frozen on dry ice and stored overnight. The following day, the cells were thawed in an ice-water bath and resuspended in 50 mL of binding buffer (5 mM imidazole, 0.5 M NaCl, 5 mM β-mercaptoethanol, 20 mM Tris pH 7.2) supplemented with an EDTA-free protease inhibitor cocktail. Cells were lysed by two rounds of French press, and the soluble fraction was collected by centrifugation at 10,000 × *g* for 10 min at 4°C.

For co-purification, the soluble material was clarified by an additional centrifugation at 18,000 rpm for 10 min at 4°C, followed by a 20-minute incubation at 37°C. In the meantime, 1 mL of Ni-NTA resin was equilibrated with 50 mL of binding buffer for 20 min. The equilibrated Ni-NTA resin was then added to the samples, and the mixture was incubated at 4°C for 2 h to allow His6-tagged proteins to bind to the resin. Following incubation, the resin was transferred to a column and washed at room temperature by gravity flow using the following steps: three rounds of 5 mL binding buffer, two rounds of 5 mL Wash Buffer 1 (40 mM imidazole, pH 7.9; 1.0 M NaCl; 20 mM Tris, pH 8.0; 5 mM β-mercaptoethanol); one round of 10 mL Wash Buffer 1; one wash with 1.5 mL High Salt Wash Buffer (40 mM imidazole, pH 7.9; 1.5 M NaCl; 20 mM Tris, pH 8.0; 5 mM β-mercaptoethanol; 5 mM CHAPS); and three rounds of 2.5 mL Wash Buffer 1. His6-tagged PrkA and its interacting proteins were eluted in three 500 µL aliquots of elution buffer (20 mM Tris, pH 7.5; 300 mM imidazole, pH 7.9; 500 mM NaCl; 5 mM β-mercaptoethanol). The eluted samples were analyzed by SDS-PAGE and sent to the NIAID Research Technology Branch (RTB) core facility for protein identification via mass spectrometry.

### MS/MS data analysis

Mass spectrometry data were analyzed using Proteome Discoverer v3.0 SP1 (Thermo Fisher Scientific) with the Byonic search engine (Protein Metrics). Spectra were searched against a composite database consisting of the *Bacillus anthracis* Sterne 34F2 proteome (UniProt proteome ID UP000031875) and a database of common laboratory contaminants (cRAP.fasta, theGPM.org). Data were filtered at a 1% peptide and protein false discovery rate (FDR) using a decoy database approach and the Percolator algorithms as implemented within Proteome Discoverer. A minimum of two peptides per protein was used for the search. Intensity-based abundance was calculated using the top three most abundant unique peptides approach. Normalization was performed using a Protein List of identifications from the corresponding negative control (BA WT::pAMY1). Common contaminants, including keratins and trypsin, were excluded from downstream analysis. Proteins are ranked based on log_2_ abundance ratios (6.64 is set as a maximum). A 1e+6 abundance cutoff in either experimental and BA WT::pAMY1-*prkA*-TEV-His6 (experimental) or BA WT::pAMY1 (negative control) sample was applied. The proteins enriched in the experimental sample were shortlisted based on the following cutoffs: abundance ratio > 2 or abundance ratio (log_2_) > 1.

### Pathway enrichment analysis

ShinyGO 0.81 (27) was used to do pathway enrichment analysis of the BAS0518 interacting proteins. UniProt was used as the pathway database, with species name set to *B. anthracis* str. “Ames Ancestor” (Accession number NC_007530). An FDR cutoff of 0.05 was used to identify significantly enriched pathways.

### Statistical analysis

Graphs and statistical analysis were performed using GraphPad Prism. *P*-value is calculated using two-tailed Student’s *t*-test.

## SUPPLEMENTAL MATERIAL

Supplemental figures jb.00518-25-s0001.docx Figures S1 to S5.

Table S1 jb.00518-25-s0002.xlsx Proteins identified as interactome of PrkA in *Bacillus anthracis*.

Table S2 jb.00518-25-s0003.xlsx Fold-enrichment pathway analysis.

ASM does not own the copyrights to Supplemental Material that may be linked to, or accessed through, an article. The authors have granted ASM a non-exclusive, world-wide license to publish the Supplemental Material files. Please contact the corresponding author directly for reuse.
