# Supplementary material for: A novel AAA+ ATPase required for sporulation and stress response in Bacillus anthracis
Source: J Bacteriol. 2026 Feb 19;208(3):e00518-25. doi: 10.1128/jb.00518-25 (PMC13001217; doi:10.1128/jb.00518-25)
Supplement: Supplemental figures — Figures S1 to S5. [file jb.00518-25-s0001.docx]

**Supplementary figures**

**A novel AAA+ ATPase required for sporulation and stress response in *Bacillus anthracis***

Nitika Sangwan^1,2#^, Ankur Bothra^2#^, Andrei P. Pomerantsev^2^, Aakriti Gangwal^3^, Rasem Fattah^2^, Mahtab Moayeri^2^, Qian Ma^2^, Sundar Ganesan^4^, Chetkar Chandra Keshavam^3^, Renu Baweja^5^, Uma Dhawan^1,*^, Stephen H. Leppla^2,*^, Yogendra Singh^3,6,*^

# Authors contributed equally

^1^Department of Biomedical Science, Bhaskaracharya College of Applied Sciences, University of Delhi

^2^Microbial Pathogenesis Section, Laboratory of Parasitic Diseases, National Institute of Allergy and Infectious Diseases, Bethesda, MD, USA

^3^Department of Zoology, University of Delhi, Delhi, India,

^4^Biological Imaging Section, Research Technologies Branch, National Institutes of Allergy and Infectious Diseases, National Institutes of Health, Bethesda, MD, USA.

^5^Department of Biochemistry, Shivaji College, University of Delhi

^6^Delhi School of Public Health, Institution of Eminence, University of Delhi

^*^Corresponding Authors: Yogendra Singh, Delhi School of Public Health, Institution of Eminence, University of Delhi, India; Email ID: [ysinghdu@gmail.com](mailto:ysinghdu@gmail.com)

Stephen H. Leppla, Microbial Pathogenesis Section, Laboratory of Parasitic Diseases, NIAID, NIH, Bethesda, USA; Email ID: [sleppla@niaid.nih.gov](mailto:sleppla@niaid.nih.gov)

Uma Dhawan, Department of Biomedical Science, Bhaskaracharya College of Applied Sciences, University of Delhi, India; Email ID: [uma.dhawan@bcas.du.ac.in](mailto:uma.dhawan@bcas.du.ac.in)

**
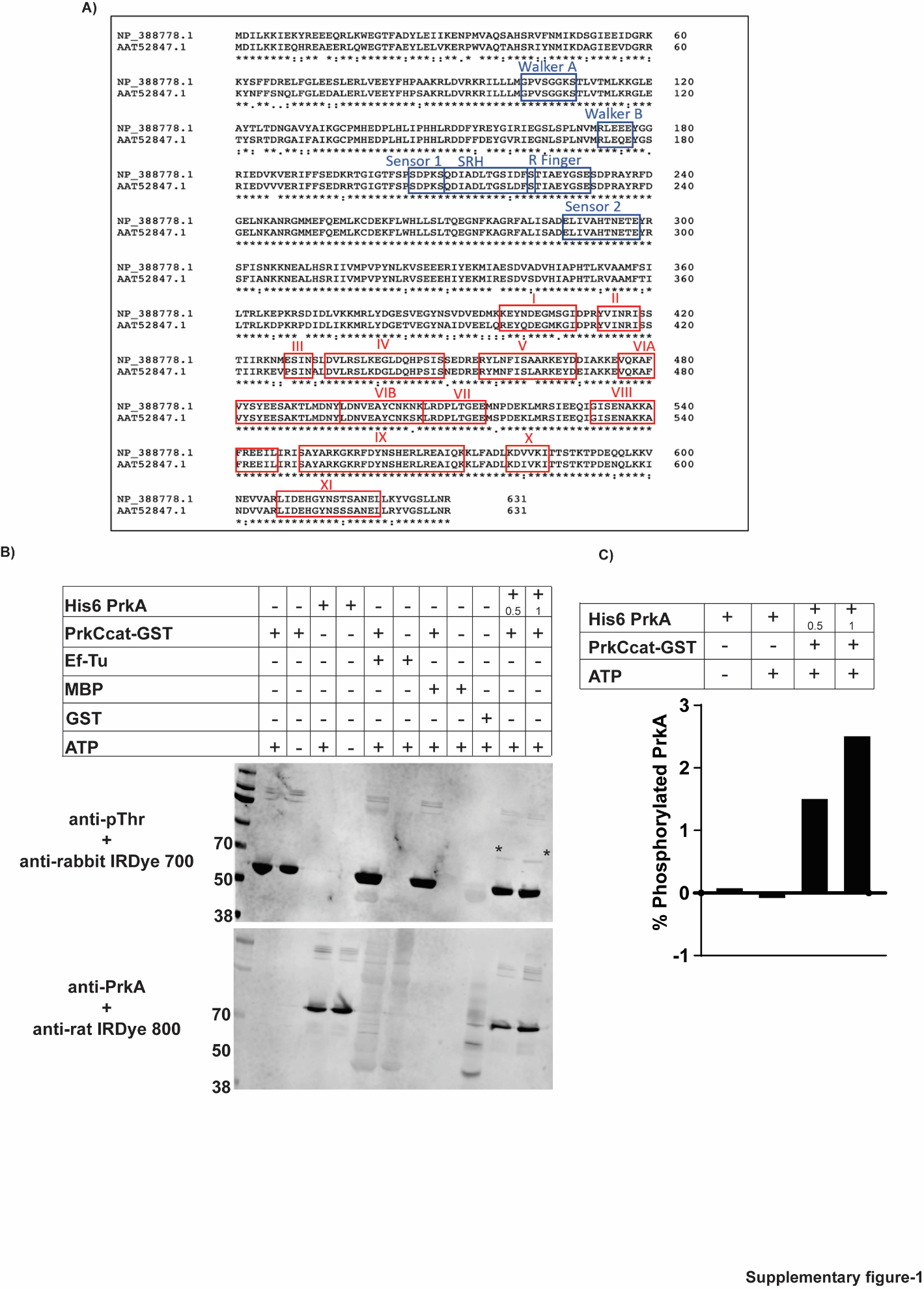
**

**Supplementary figure 1: A)** Pairwise sequence alignment between BA PrkA and *Bacillus subtilis* PrkA showing multiple conserved motifs- blue boxes highlight the ATPases domains (AAA+) in the N-terminal part of the two proteins. Red boxes highlight the Hanks-type kinase domains in the C-terminal part of the proteins. NP_388788.1- denotes protein sequence of *Bacillus subtilis* and AAT52847.1- denotes protein sequence of *Bacillus anthracis*. **(B)** Immunoblot analysis showing threonine phosphorylation of His6 PrkA (0.5 µg) by the GST-tagged catalytic domain of PrkC (PrkCcat-GST, 0.5 µg), detected using an anti-phosphothreonine (anti-pThr) antibody and probed with anti-rabbit IRDye 700 (B). Phosphorylated PrkA is indicated with an asterisk (*). The amount of His6 PrkA loaded in each lane was monitored using an anti-PrkA antibody, probed with anti-rat IRDye 800. Purified EF-Tu (5 µg), MBP (10 µg), and GST (5 µg) proteins were used as positive and negative controls for the anti-pThr activity of PrkCcat-GST. Last lane has 1 µg of His6 PrkA. **(C)** Bar graph representing % phosphorylated PrkA, measured as the pixel intensity of bands detected with the anti-pThr antibody, normalized to the pixel intensity of the corresponding bands detected with the anti-PrkA antibody.

**
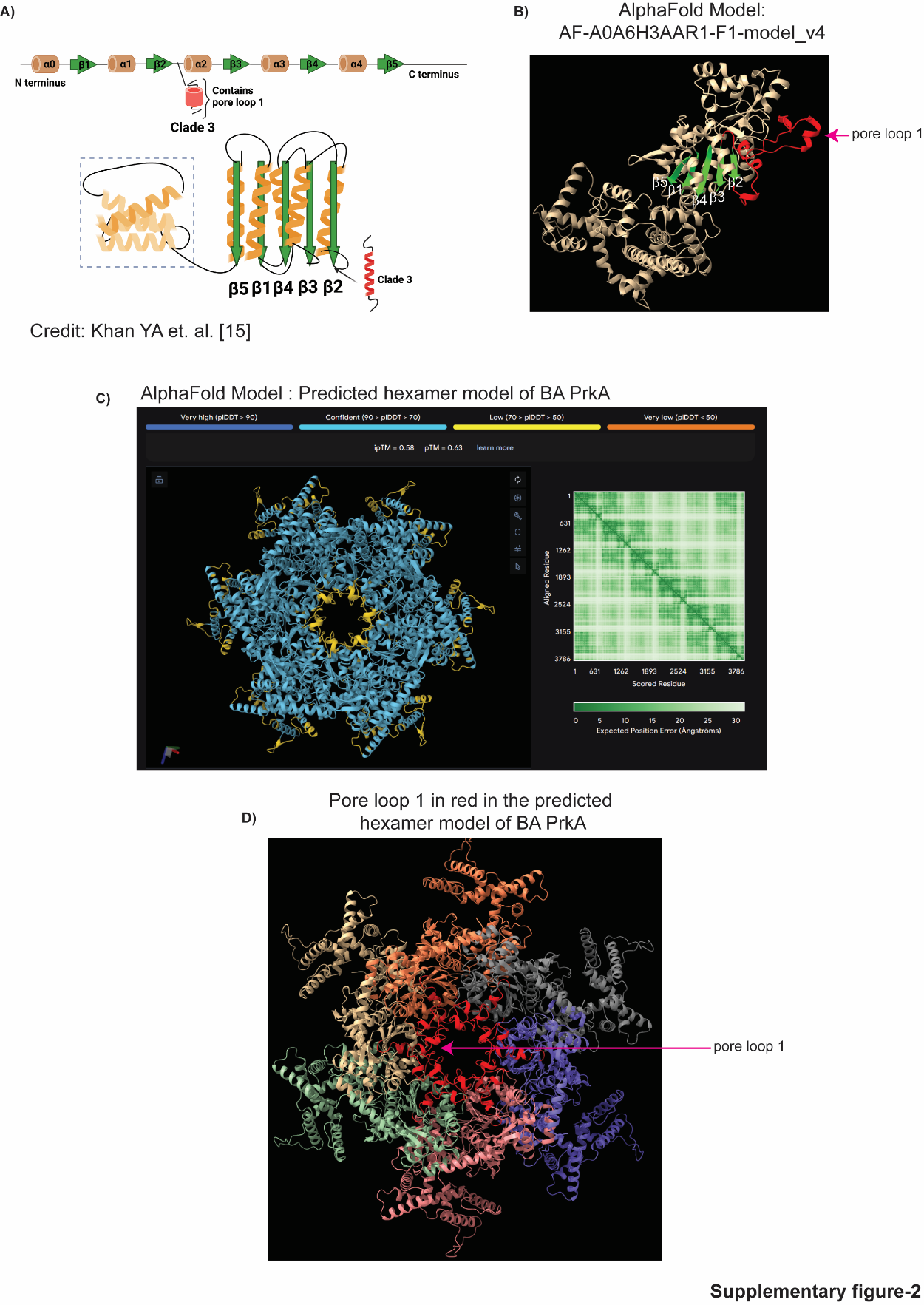
**

**Supplementary figure 2: (A)** Classification basis of AAA+ superfamily Clade III [23]. Clade III of AAA+ superfamily has a unique pore loop 1 between β2 and β3 of the core AAA+ domain. **(B)** AlphaFold 3 predicted structure of BA PrkA (AF ID: AF-A0A6H3AAR1-F1-model_v4). The core AAA+ domain is coloured lime and the extended pore loop 1 is coloured red. **(C)** Predicted model of hexameric BA PrkA. The pIDDT value indicates a statistically strong structural prediction. **(D)** The predicted hexameric ring of BA PrkA, with pore loop 1 coloured red.


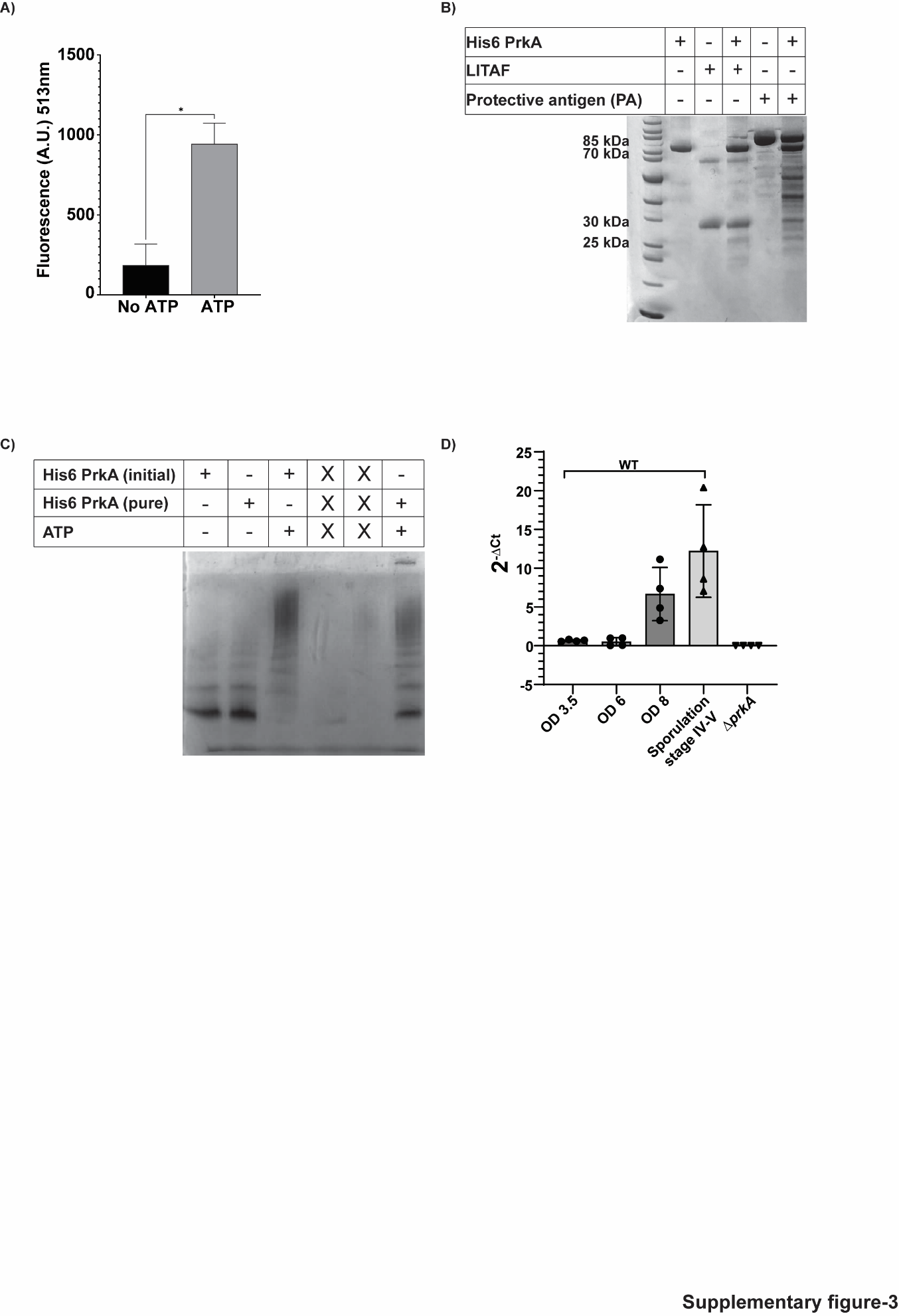


**Supplementary figure 3: A)** Bar graphs depicting protease activity of His6 PrkA (with amido-hydrolase contaminant) evaluated by cleavage of fluorescently labelled BODIPY FL-Casein at 2 hours in the absence and presence of ATP respectively. N = 3 experiment. **(B)** Coomassie stained SDS-PAGE depicting proteolysis of LITAF and PA using His6 PrkA (with amido-hydrolase contaminant) with 24 hours incubation at 37°C. Control lanes have His6 PrkA and respective substrates (LITAF and PA) incubated at 37°C for 24 hours for background resulting from autohydrolysis of respective proteins. His6 PrkA and substrates were used at concentrations of 0.1µg/µl. N = 5 experiments. **C)** Coomassie stained Nativ-PAGE depicting oligomeric state of His6 PrkA purified in two forms as discussed in results section. 2µg of both forms (initial and pure form) were resolved in 8% Native Page. Lane marked X has no proteins. **D)** RT-qPCR analysis represented as bar graph confirming the BA *prkA* expression at mentioned ODs during vegetative growth of bacteria in nutrient-rich LB media. Expression of BA *prkA* under Sporulation stage IV-V was used as the positive control and in BA *ΔprkA* strain as the negative control. The average values of genes relative to the housekeeping gene *rpoB* is represented as 2^-Δct^ ± standard deviation (SD).


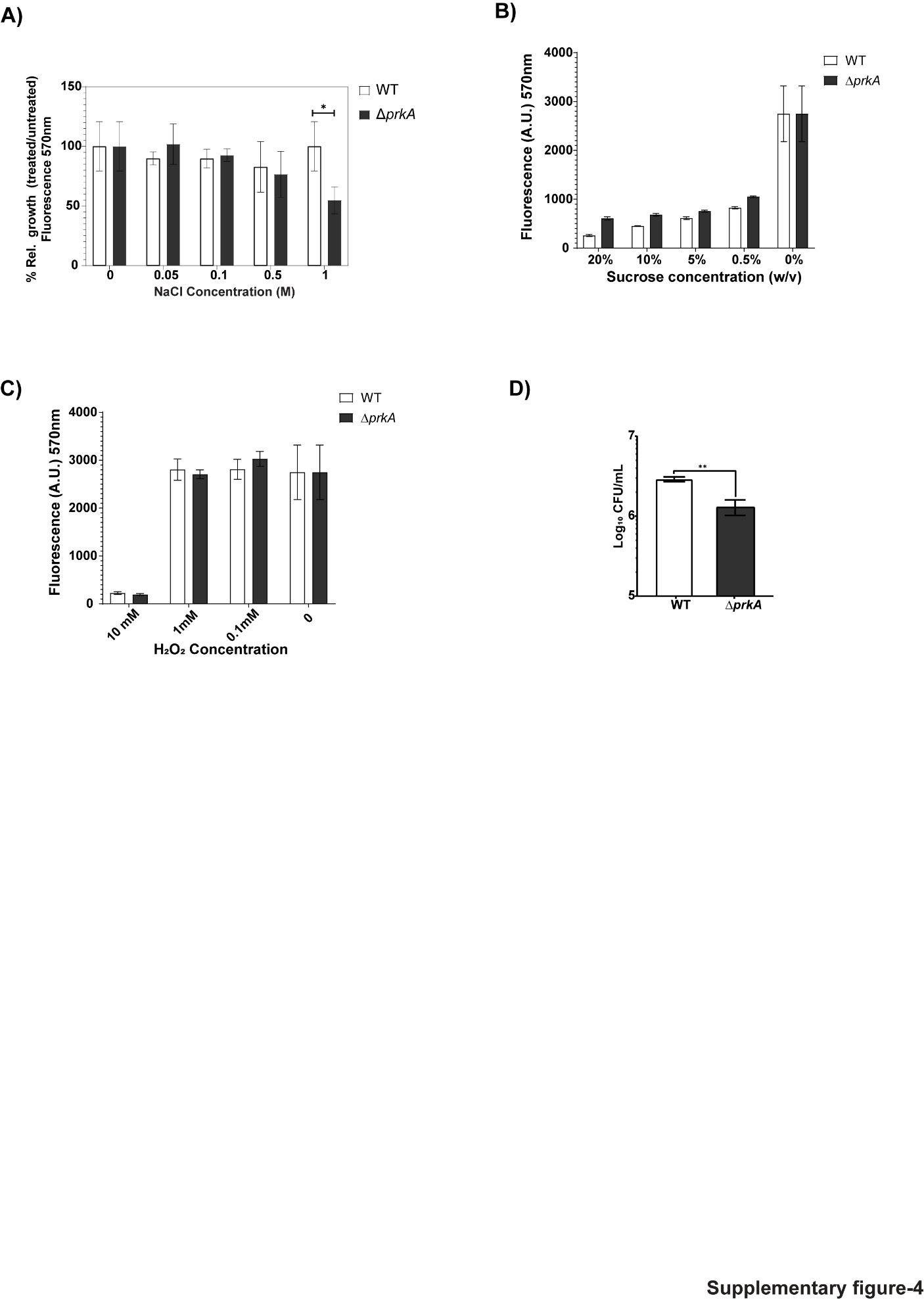


**Supplementary figure 4:** (**A**) Bar graph representing mean ± SD of the percent relative growth (y-axis) of BA WT (white bar) and BA *ΔprkA* (black bar) strains in LB medium supplemented with varying concentrations of NaCl (0M, 0.05M, 0.1M, 0.5M, and 1M). Growth is represented as a percentage of the maximum growth observed in the respective concentration of NaCl (x-axis), with values normalized to growth under no-salt conditions. N = 2 experiments. (**B**) non-ionic osmotic, and (**C**) oxidative stress generated using different concentrations of sucrose and H_2_O_2_, respectively. N = 3 experiments. (**D**) Bar graph showing mean ± SD of colony-forming units per ml (CFU/ml) of BA WT (white bar) and BA *ΔprkA* (black bar) strains surviving in LB medium supplemented with 1 M NaCl. N = 2 experiments.


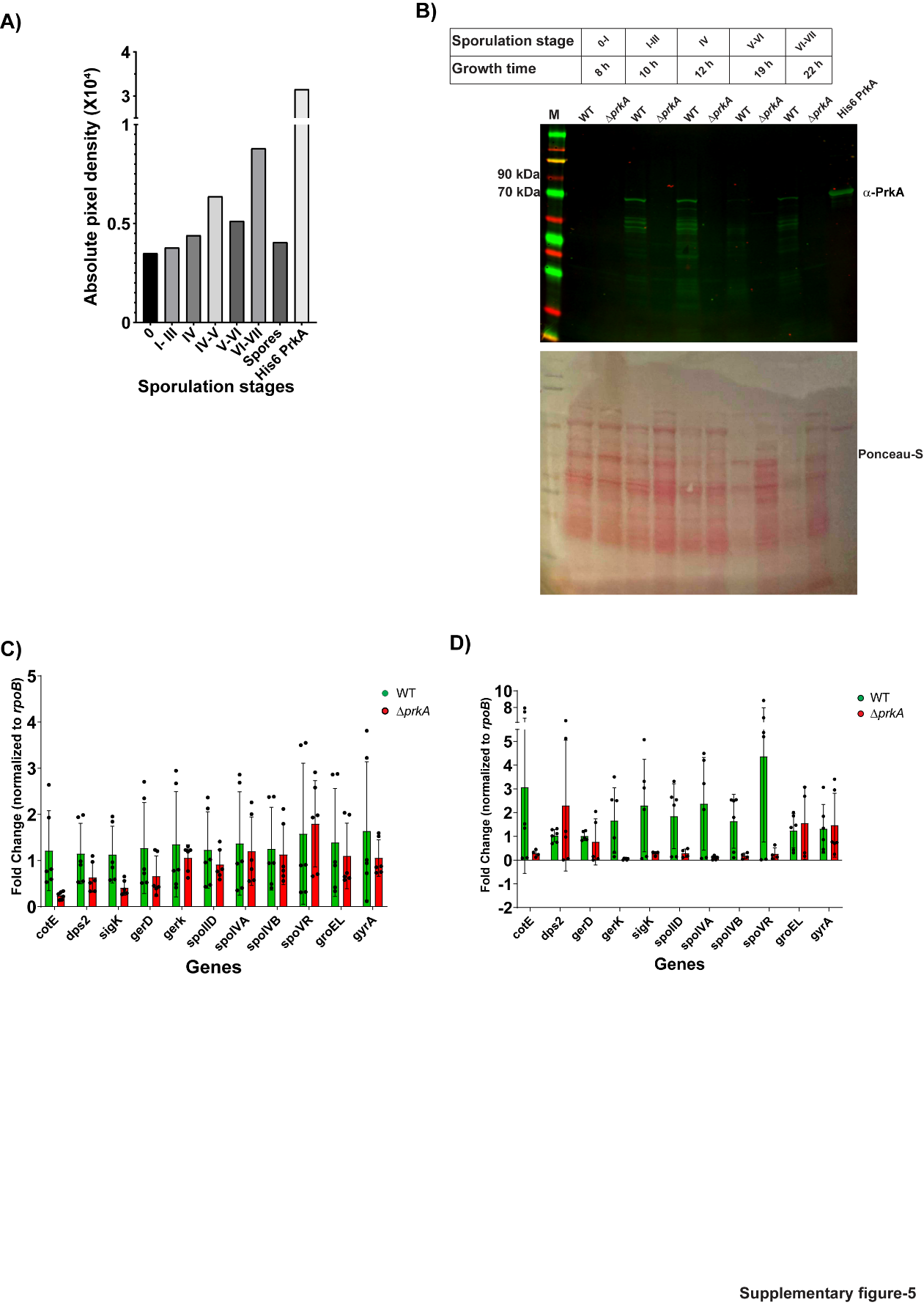


**Supplementary figure 5: (A)** Bar graph depicting densitometric analysis of Figure-3B as absolute pixel density of BA PrkA protein from at different stages of sporulation. **(B)** Immunoblot with anti-PrkA showing temporal expression of BA PrkA protein at various time points corresponding to different stages of sporulation in BA WT and BA *ΔprkA*. Growth time and corresponding sporulation stages are mentioned on the top. Lane M: protein ladder, lane 12: purified His6 PrkA protein (positive control). Data are representative of N = 2 experiments. Protein in each lane is normalized to total protein concentration estimated using BCA method. **(C, D)** RT-qPCR analysis represented as bar graphs showing the relative RNA expression of various sporulation-related genes during early (**C**, Stage 0–III, growth time 8h) and late (**D**, Stage V–VI, growth time 19h) stages of sporulation. Expression levels were normalized to housekeeping gene *rpoB*. Data represent three independent experiments (N = 3). Green bars represent the wild-type (BA WT) strain, and red bars represent the BA *ΔprkA* mutant. Individual data points are shown as filled circles for both strains.
